# Striking the Right Chord: Signaling Enigma during Root Gravitropism

**DOI:** 10.3389/fpls.2017.01304

**Published:** 2017-07-27

**Authors:** Manjul Singh, Aditi Gupta, Ashverya Laxmi

**Affiliations:** ^1^National Institute of Plant Genome Research New Delhi, India; ^2^Interdisciplinary Centre for Plant Genomics, University of Delhi South Campus New Delhi, India

**Keywords:** Arabidopsis, gravitropism, phytohormones, glucose, signaling, root

## Abstract

Plants being sessile can often be judged as passive acceptors of their environment. However, plants are actually even more active in responding to the factors from their surroundings. Plants do not have eyes, ears or vestibular system like animals, still they “know” which way is up and which way is down? This is facilitated by receptor molecules within plant which perceive changes in internal and external conditions such as light, touch, obstacles; and initiate signaling pathways that enable the plant to react. Plant responses that involve a definite and specific movement are called “tropic” responses. Perhaps the best known and studied tropisms are phototropism, i.e., response to light, and geotropism, i.e., response to gravity. A robust root system is vital for plant growth as it can provide physical anchorage to soil as well as absorb water, nutrients and essential minerals from soil efficiently. Gravitropic responses of both primary as well as lateral root thus become critical for plant growth and development. The molecular mechanisms of root gravitropism has been delved intensively, however, the mechanism behind how the potential energy of gravity stimulus converts into a biochemical signal in vascular plants is still unknown, due to which gravity sensing in plants still remains one of the most fascinating questions in molecular biology. Communications within plants occur through phytohormones and other chemical substances produced in plants which have a developmental or physiological effect on growth. Here, we review current knowledge of various intrinsic signaling mechanisms that modulate root gravitropism in order to point out the questions and emerging developments in plant directional growth responses. We are also discussing the roles of sugar signals and their interaction with phytohormone machinery, specifically in context of root directional responses.

## Events During Graviresponse in Plants

The gravitropic response mechanism can be divided into several sequential components, including perception of the change in the gravity vector, transduction, and asymmetrical growth response. Unlike unilateral light, gravity does not form a gradient between the upper and lower sides of an organ. All parts of the plant experience the gravitational stimulus equally. The first step of gravitropism addresses how do plant cells detect gravity? Two hypotheses have been proposed to explain how the direction of gravity is perceived by plants: (1) the gravitational-pressure model and (2) the starch-statolith hypothesis. The latter has been strongly supported by a variety of experimental approaches in various plant species. The second component of the gravitropic response mechanism is transduction, in which the development of hormone asymmetry is obtained. In the third step, a curvature response is established that allows the organ to resume growth at a defined set angle from the gravity vector; the gravitational set point angle (GSA). GSA is defined as the angle with respect to the gravity vector at which an organ is maintained as a result of gravitropism (Digby and Firn, 1995). The gravity vector (GSA = 0°) helps decide the GSA values of different organs, if an organ is maintained vertically and grows downward towards gravity vector it has a GSA of 0° (e.g., a primary root) while an organ growing vertically upward against the gravity vector will have a GSA of 180° (e.g., a primary shoot). Any organ growing at non-vertical angles will have a GSA between these two extremes (Digby and Firn, 1995). For a plant organ to guide its growth along a defined GSA, it must perceive any change in its orientation within the gravity field.

### Gravity Perception

**FIGURE 1:**
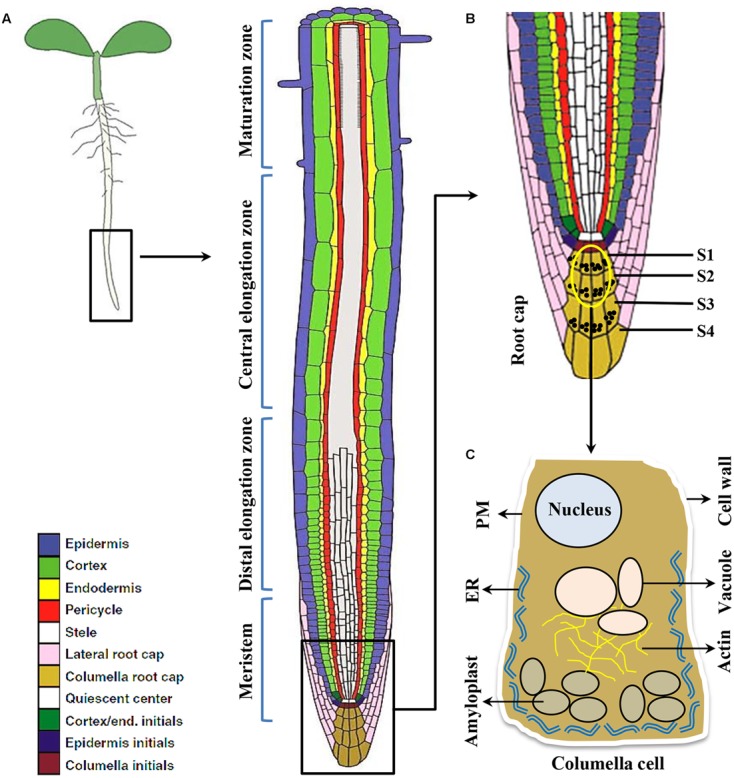
The root cell types responding to gravity stimulus in *Arabidopsis*. **(A)** Longitudinal view of an Arabidopsis primary root showing meristem, Distal and central elongation zones, maturation zone. **(B)** Gravity sensing in roots occurs in the central columella cells of the root cap (inset). The root cap consists of four layers of cells important for root gravitropic response namely S1, S2, S3, S4, respectively. However, the central columella cells from S1 and S2 (the encircled cells) play major role for gravity sensing. **(C)** Columella cell (cartoon) contains starch-filled organelles called amyloplasts, nucleus, vacuole, actin filaments, endoplasmic reticulum (ER), plasma membrane (PM), and cell wall.

**FIGURE 2:**
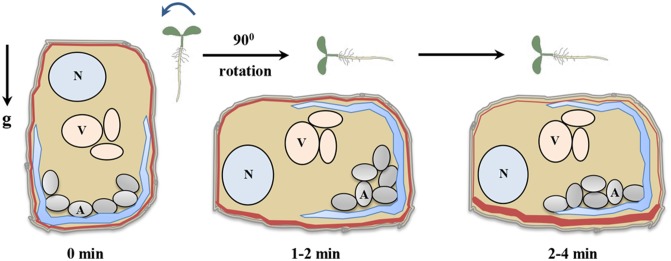
Gravity sensing in columella cells. Roots when grown vertical have been designated time 0 min. Reorientation of seedling by rotating to 90° generates a gravistimulus and initiate gravisensing process. The amyloplasts start to settle towards the new bottom of columella cells immediately post reorientation and within 2–4 min of reorientation, amyloplast settles down completely (Leitz et al., 2009). The amyloplast sedimentation creates pressure on endoplasmic reticulum membrane as a result activating mechanosensitive sites (Monshausen et al., 2011). After 2 min of reorientation, PIN3 proteins start to relocalize thereby regulating auxin flow (Friml et al., 2002). N (nucleus), V (vacuole), A (amyloplast), g (gravity), and red lines indicate PIN3 distribution.

Plant roots are simple structure divided into various sections like root cap, meristem, elongation zone and maturation zone (**Figure 1**). The root cap protects root tip from obstacles in the soil and at the same time act as a guiding sensor for directional growth. There are usually four layers of columella cells at the root tip named S1-S4 (towards root tip), S1 and S2 are important for root gravitropism (Blancaflor et al., 1998). Amyloplasts that function as gravity sensors are called statoliths. These statoliths are of sufficiently high density relative to the cytosol thereby readily sediment to the bottom of the cell. The specialized gravity-sensing cells in which statoliths occur are called statocytes. The statocytes are cells with small vacuoles and a cortical ER, also their nucleus is positioned toward the shoot-ward side (Leitz et al., 2009) (**Figure 2**). Cortical microtubules and actin microfilaments contribute to development and maintenance of this polarity, whereas the lack of endoplasmic microtubules and prominent bundles of actin microfilaments probably facilitates sedimentation of statoliths (Volkmann and Baluska, 1999).

According to the starch-statolith hypothesis (Haberlandt, 1900) gravity reorientation relocates the amyloplasts to the new basal side of the cells (**Figure 2**). Sedimentation of amyloplasts towards gravity vector acts as a signal to plants which is then translated to biochemical signal that evoke the gravitropic response. In literature, the starch-statolith hypothesis of gravity sensing is supported extensively (Morita, 2010). Investigations on starchless mutant *phosphoglucomutase* (*pgm*) of Arabidopsis lacking a starch synthesis enzyme showed that they are still gravitropic but their gravity response was strongly reduced (Kiss et al., 1996). Also, ablation of columella cells through lasers or genetic means greatly reduces graviresponse (Blancaflor et al., 1998; Tsugeki and Fedoroff, 1999). Support for starch-statolith theory also comes from the use of high gradient magnetic fields to displace statoliths. Magnetophoretic displacement of statoliths causes the roots to curve toward the direction of magnetic field while hypocotyls move away from the magnetic field (Kuznetsov and Hasenstein, 1996, 1997). Changes in density of amyloplasts also affect graviresponse, the *sex1* (*starch excess*) mutant in Arabidopsis, which is impaired in degradation of starch, accumulates excess starch in various tissues (Vitha et al., 2007). The gravitropic sensitivity of *sex1* mutant roots was comparable to that of wild type (WT) roots. However, in hypocotyls, the *sex1* mutant was much more sensitive towards gravity stimulus as compared to the WT (Vitha et al., 2007). Recently, a study on gravity induced distribution of DII-Venus in *pgm1* mutant provided a link between statolith sedimentation and auxin redistribution (Band et al., 2012). Also, a sudden change in amyloplast sedimentation was shown to cause differential distribution of auxin on either side of the root (Baldwin et al., 2013). These results confirm a crucial role of statoliths as a sensor of gravistimuli in plants. The elongation zone is also a critical region for studying directional movements because it is highly plastic in nature and during gravitropism, when seedlings are oriented towards their side, the visible bending is observed in the elongation zones (Miller et al., 2007). The anatomy and morphology of the graviresponding organs indicates a spatial separation between signal perception, signal formation, signal transduction and differential growth (Tasaka et al., 1999). Simple bending curvature of plants in response to change in direction of gravity vector thus becomes a complicated process.

Apart from the support provided for starch-statolith theory, there are enough clues for the existence of alternate mechanisms of gravity sensing in plants. These secondary mechanisms are supposed to be independent of starch and might also govern gravitropic bending. For example, the *pgm* mutants, having greatly attenuated gravitropic response, could eventually reorient their roots downward and stems upwards. (Mullen et al., 2000) have used a ROTATO device to study gravitropic response; this device holds selected region of roots at specific angles from the gravity vector on a motorized stage with an automated camera attached to it. ROTATO allows the root cap of a graviresponding root to maintain vertical non-stimulated orientation while any selected region within the elongation zone can be maintained at a gravistimulated angle. The *pgm* mutants responded at constant rate regardless of the increase in angles, whereas the response of WT roots increased when constrained at greater angles (Wolverton et al., 2011). The *pgm* mutants also lacked the auxin gradient formation as visualized by DR5 reporter expression (Wolverton et al., 2011). This result suggests that, statolith sedimentation is not the only mode of gravity sensing and there is some unknown mechanism that triggers the residual root gravitropic response in the *pgm* mutant independent of the angle of tip orientation. ROTATO experiments have also shown that 20% of the gravity response comes from the region within the distal elongation zone and not the root tip (Wolverton et al., 2002). ROTATO can be used as an advantageous instrument to study root gravitropism as it facilitates the exposure of any section of roots at any angle not only to microgravity conditions but also to hypo-gravity conditions with some modifications (Ishikawa et al., 2007). Removal of root cap reduces the root gravitropic response but doesn’t abolish it. However, roots whose root cap is removed respond differently to gravistimulation in the presence of actin-polymerization-inhibiting drugs and these decapped roots showed faster gravi-bending as compared to WT (Mancuso et al., 2006). These results suggest that roots can sense gravity outside the zone of root cap which also depends on actin. As roots, do not contain sedimenting amyloplasts beyond the columella cells, therefore, the starch-statolith model might not be the only mode of gravisensing in plants (Baldwin et al., 2013). Studies on green algae *Chara* has provided the alternative hypothesis of gravisensing, i.e., the protoplast pressure hypothesis. Since, *Chara* do not contain statoliths and uses gravity as a signal to regulate their cytoplasmic streaming (Wayne et al., 1990; Staves, 1997). More studies are awaited to validate these findings and support different gravisensing hypothesis. Since root cap is major site of gravity perception, there should be some mean of communication between the gravity-sensing cells of root cap and the cells present back in the root elongation zone that ultimately respond to the gravity signal in roots.

### Gravity Induced Signals in Plants

Gravity is the only constant factor, both in direction and magnitude, to which plants definitely need to- and have to- adapt. Post gravity perception, a series of events takes place to transduce the signal within the plant system. With the use of electron tomography, it was found that, in response to gravity stimulus, sedimenting amyloplast can bend, and distort the endoplasmic reticulum (ER) upon contact (Leitz et al., 2009). The distortion of ER possibly opens stretch-activated mechano-sensitive ion channels. The other hypothesis suggests a possibility of protein–protein interactions between the molecules attached to the amyloplasts and the ER due to close contact between both of them (Leitz et al., 2009). Signal transduction in gravitropism employs variety of signaling components and second messengers such as Ca^2+^ and pH (Fasano et al., 2001), actin (Blancaflor, 2013), inositol 1,4,5-triphosphate (InsP3) (Perera et al., 2006) etc. However, all these scattered findings don’t give clear picture about a comprehensive pathway for gravitropism.

According to the mechano-sensitive ion channel hypothesis of signal transduction, the falling amyloplast create pressure on ER or plasma membrane either directly or via actin filaments. The pressurized ER membrane opens mechano-sensitive ion channels leading to changes in concentration of ions, such as Ca^2+^ which in turn leads to repolarization of statocytes, relocalization of PINs and subsequent changes in auxin transport (Baldwin et al., 2013). Gravity induced changes in Ca^2+^ levels are too minute to be detected. Attempts to observe changes in Ca^2+^ levels in the statocytes with the use of indo-1 as the calcium sensitive detection system have not been successful in past (Legué et al., 1997). It is found that calmodulins are highly expressed in columella cells of roots (Stinemetz et al., 1987); this can be correlated with the fact that though undetected, small gravity-induced changes in intracellular Ca^2+^ levels could affect cell signaling. Recent advancement in sensors have helped in Ca^2+^ imaging technology and the detection of cytoplasmic Ca^2+^ changes in response to gravistimulation in both roots and shoots (Toyota and Gilroy, 2013). With the use of genetically encoded Yellow Cameleon (YC) 3.6 Ca^2+^ biosensor, it was revealed that after gravistimulation, a wave of Ca^2+^ arises on the lower side of the root toward the elongation zone similar to auxin (Monshausen et al., 2011). Auxin alone is found to induce Ca^2+^ levels and signals (Toyota and Gilroy, 2013). Further it is known that the increase in Ca^2+^ levels is able to regulate auxin transport through regulation of PINOID (PID) kinase (Robert and Offringa, 2008). In addition, asymmetrical application of Ca^2+^ through agar block, caused root bending toward the Ca^2+^ source (Lee et al., 1983a). The relevance of Ca^2+^ signals upon gravistimulation is further substantiated with the fact that gravitropic bending is severely impaired upon chelation of apoplastic Ca^2+^ or by inhibition of calmodulin or Ca^2+^ channels (Lee et al., 1983b; Vanneste and Friml, 2013). Use of auxin transport inhibitors such as triiodobenzoicacid (TIBA) or 1-*N*-naphthylphthalamic acid (NPA) has been found to block the gravity-induced Ca^2+^ redistribution in maize and pea roots (Lee et al., 1984). Additionally, Ca^2+^ dependent cross-linking of the acidic groups in pectin, Ca^2+^ dependent regulation of enzymes and Ca^2+^ modulated proteins calmodulins are also found in the extracellular matrix (Toyota and Gilroy, 2013). These findings suggest a critical role of extracellular Ca^2+^ in modulating gravitropic growth. Further studies are required to know others players regulating Ca^2+^ pumps and channels that drive these apoplastic fluxes and how amyloplast mediated perception system regulates these components.

Post gravistimulation, changes in pH also take place in columella cells. Ca^2+^ levels cause alterations in cell wall pH which can regulate elongation via acid growth (Monshausen et al., 2011). Changes in pH affect many cellular activities such as enzyme function, hormone distribution etc. Use of chemical inhibitors such as; benzoic acid, bafilomycin A1 (a vacuolar H^+^ATPase inhibitor) or use of caged protons to block pH changes caused reduction in root gravitropism but not abolished it (Scott and Allen, 1999; Fasano et al., 2001). This gives us the idea that gravistimulated changes in pH might also regulate the efficiency of signals generated within the gravity perceiving columella cells. In gravistimulated cells, change in pH can be linked with relocalization of the PIN proteins since the mutants defective in gravity induced columella cell alkalization fail to show PIN relocalization (Boonsirichai et al., 2003; Harrison and Masson, 2008).

Mechano-sensitive channels could also open due to straining of actin filaments caused by amyloplast sedimentation as the amyloplasts are usually surrounded by a network of actin filament in the columella cells (Collings et al., 2001; Yoder et al., 2001). Surprisingly, actin destabilizing drugs like latrunculin B (Lat-b) and cytochalasin D promotes gravitropism in roots rather than inhibiting it, this way actin acts a negative regulator of gravity sensing (Blancaflor et al., 2003; Hou et al., 2004). Disruption of actin could possibly lead to more rapid sedimentation of amyloplast causing a higher compressive force on the ER, thereby adding to the overall gravity sensing and signaling potential (Yoder et al., 2001; Blancaflor, 2013; Zheng et al., 2015). With the use of microrheology analyses, amyloplast sedimentation has been studied extensively and it was found that there is abundance of actin-cytoskeletal network in the intracellular regions of central columella cells which determines the dynamics of falling amyloplasts (Zheng et al., 2015). Analysis of *distorted1* (*dis1*) mutant whose functional protein encodes for the ARP3 subunit of the Arabidopsis Actin-Related Protein 2/3 (ARP2/3) complex revealed that *dis1* mutant has disorganized thick actin bundles and showed delayed root bending upon gravity reorientation (Zou et al., 2016). The *dis1pgm1* double mutant showed more delayed gravitropic curvature which was not enhanced upon Lat-B treatment suggesting that DIS1 is involved in gravity sensing as well as signal transduction (Zou et al., 2016). Further, it was found that ARP3/DIS1 was involved in asymmetric auxin redistribution via modulation of PIN cycling (Zou et al., 2016).

Possible physical interactions between the sedimenting amyloplast and the ER bound proteins can also be involved in generating gravity signals within cells. The ligand-receptor hypothesis came from the study of single-celled rhizoids of the green algae *Chara*. In *Chara*, the statoliths are barium-sulfate-filled vesicles. During parabolic flights the weightless statoliths were able to trigger gravity signal as long as there was a contact with sensitive sites in plasma membrane (Limbach et al., 2005). It was then proposed that, in *Chara* rhizoids, pressure exerted by statoliths weight was not responsible for gravity sensing, rather it was dependent upon direct contacts between components present on statoliths and membrane-bound receptors (Braun and Limbach, 2006). Similar type of phenomenon may also exist in plants and can be correlated with the fact that reduced gravitropic sensitivities in starchless mutant can be restored by sedimenting their plastids through hypergravity (Fitzelle and Kiss, 2001). This hypothesis was further substantiated with the screens for various mutants showing altered gravitropic responses. The Arabidopsis mutant *altered response to gravity1 (arg1)* showed reduced root and hypocotyl gravitropism (Sedbrook et al., 1999). The *ARG1* gene encodes a DNA-J like protein with conserved J domain and a coiled-coil region (Sedbrook et al., 1999). *ARG1* has two paralogs; *ARG-like1* (*ARL1*) and *ARL2*, of which *arl2* mutants showed gravitropic defects very similar to *arg1*. Both ARG1 and ARL2 operate in the same pathway. ARG1 localizes to the endomembrane system, whereas ARL2 is mainly found associated with the plasma membrane and selectively expressed in gravisensing cells (Boonsirichai et al., 2003; Harrison and Masson, 2008). To find out genetic enhancers of *arg1*, researchers used forward genetics approach and isolated two mutants; *modifier of arg1* (*mar1*) and *mar2*. MAR1 and MAR2 belong to the Translocon of the Outer envelope of Chloroplasts (TOC) complex and encode for two different components TOC75 and TOC132, respectively, in Arabidopsis (Stanga et al., 2009). The TOC complexes consist of a pore (Toc75/MAR1); Toc159 family receptors (TOC159, TOC132/MAR2, TOC120, or TOC90); and a TOC34 family receptor (TOC33 or TOC34/PPI3). The TOC complex helps in delivery of nuclear encoded proteins to chloroplasts (Strohm et al., 2014). ARG1 and the TOC complex have different functions and are differentially localized. The *mar2* single mutant has no obvious gravitropic phenotype while *arg1mar2* double mutants are agravitropic (Stanga et al., 2009). The columella cell amyloplasts are also normal in *arg1mar2* double mutants; they contain normal amounts of starch and have normal sedimentation kinetics (Stanga et al., 2009). Why the corresponding mutations show a strong genetic interaction within the gravitropism signaling raises points for the ligand-receptor hypothesis. TOC132 act as a receptor on plastid surface for preprotein recognition during translocation (Perry and Keegstra, 1994). This finding fits well with the hypothesis that TOC132 would play a role in mediating plastid interactions with proteins located on the cortical ER or plasma membrane during gravistimulation (Baldwin et al., 2013).

Another signaling molecule involved in graviresponse is Inositol 1,4,5-trisphosphate (InsP3). Evidence for the role of InsP3 in gravitropism came from measurement of InsP3 levels during early gravitropic responses. Upon gravistimulation, InsP3 fluxes were found to first fluctuate and then increase at the bottom half of oat pulvinus (Perera et al., 2001). Similar results were also observed in case of Arabidopsis stem (Perera et al., 2006). The Arabidopsis root, stem and hypocotyl showed gravitropic defects upon constitutively overexpressing human TYPE I INOSITOL POLYPHOSPHATE 5-PHOSPHATASE (InsP 5-ptase) which specifically hydrolyses InsP3 (Perera et al., 2006). Activation of PHOSPHOLIPASE C (PLC) leads to an increase in inositol 1,4,5-triphosphate (InsP_3_) levels. Microarray studies conducted on plants, manipulated in their InsP3 metabolism by inhibiting PLC activity or overexpressing InsP 5-ptase, identified InsP_3_-dependent and independent co-regulated genes in response to gravity (Salinas-Mondragon et al., 2010). In Arabidopsis, *INOSITOL POLYPHOSPHATE 5-PHOSPHATASE 13* gene (*5PTase13*) encodes for an enzyme involved in breaking down of InsP3. The knockout *5pt13* mutant has higher levels of InsP3 and enhanced root gravitropic responses (Wang et al., 2009). The *5pt13* mutant shows reduced response to auxin transport inhibitor NPA, suggesting an increased polar auxin transport (Wang et al., 2009). In another study, it was found that PHOSPHATIDYLINOSITOL MONOPHOSPHATE 5-KINASE (PIP5K), a key enzyme in the phosphatidylinositol pathway, is involved in gravitropism. In phosphatidylinositol pathway, PIP5K catalyzes synthesis of PI-4,5-bisphosphate, a precursor of secondary messengers InsP3, and diacylglycerol (DAG) (Mei et al., 2012). The knockout *pip5k2* mutant shows delayed root gravity response (Mei et al., 2012). The *pip5k2* mutants are more sensitive to NPA as compared to WT, suggesting that it has impaired polar auxin transport (Mei et al., 2012). Recently, a root-specific protein named InteractoR Of SYnaptotagmin1 (ROSY1) has been identified as one of the earliest transcribed signals during root gravitropic response in Arabidopsis (Dalal et al., 2016). ROSY1 accumulates on the upper side of the gravistimulated root and *rosy1-1* mutant roots displayed faster gravitropic bending. ROSY1, is a stigmasterol binding protein which interacts with a membrane transport and recycling protein, synaptotagmin-1 and thus might affect vesicle recycling during the asymmetrical gravitropic growth (Dalal et al., 2016).

### Other Directional Responses in Plants

Root growth is governed by the coordinated events of cell division and elongation of the newly formed cells. Plants roots are extremely sensitive to environmental stimulation such as; gravity, mechanical obstacles, light, moisture and nutrient gradients modulating the directional growth of roots to obtain an optimal growth trajectory. The initial work of Darwin represented various plant movements which were defined as a result of circumnutation (Darwin and Darwin, 1880). Plant movements are categorized in various categories such as “tropic movement” induced by a constant directional factor like gravity or light and “nastic movement” induced by an external factor but independent of direction of stimulus. Circumnutation is the circular or elliptical movement attained by plant organs while growing through a particular direction. Nutations are organ movements created due to unequal growth rate on two sides of an organ and are governed mainly by internal factors (Migliaccio et al., 2013). Out of these movements, tropic and nastic movement mostly occur together in plants. These movements produce various growth patterns in roots based on the substrate and environment. Among these growth patterns produced in roots are; root-waving, -coiling and -skewing etc. When Arabidopsis seedlings are grown on slanted high agar density (1.5–2%) containing media they show waving and skewing pattern of root growth. This kind of growth behavior is a result of touch, gravity and circumnutation (Migliaccio and Piconese, 2001). Waving is not apparently seen when the agar plates are set vertically. Once the plates are slanted, root experience gravity and tries to move downward but unable to penetrate the hard medium which results in a forced response towards a touch stimulus. This cumulative stimulus of touch and gravity when accumulated with circumnutation gives rise to root-waving. However, studies evaluating the role of environment and nutritional conditions further complicated our understanding of this process (Buer et al., 2000). Another peculiar aspect of Arabidopsis root growth, normally visible in commonly used ecotypes like Landsberg (L*er*), Wassilewskija (Ws) and Columbia (Col), in which roots tend to move in a particular direction when grown vertically is termed as skewing (Simmons et al., 1995). The intensity of the slanting differs among the accessions and in some way might be connected with the waving movements. The root skewing was further studied and it was observed that the observed rotation of root tip was an outcome of epidermal cell file rotation (CFR) (Yuen et al., 2005). The slanting movement is a result of touch stimuli and twisting of Arabidopsis roots. It should be taken into account that waving and skewing are visible on the surface and do not appear when a root grows within the medium because inside the medium root experiences a uniform touch/pressure around the growing root. Skewing is quantified as rightward or leftward based on the slant angle on the medium whereas waving is usually quantified by their frequency and amplitude of waves. Coiling is another kind of movement shown by Arabidopsis roots when grown on horizontal plates. The coils formed are always right handed in WT with roots showing left handed torsion (Mirza, 1987). The morphology of coils, varies depending on the nutritional quality and conditions of illumination (Mirza, 1987). Taken together, analysis of waving, skewing and coiling in addition to tropic responses can be a good system to study the dynamics of root growth and movement, however the actual root growth movement in the soil is different and any conclusions drawn based on root-gel/medium interactions need further confirmation in the soil system.

## Phytohormones and Graviresponses in Plants

Phytohormones have profound effects on development at vanishingly low concentrations. The emerging concept of cooperative hormone action opens new possibilities for a better understanding of the complex interactions between all phytohormones and their possible synergistic effects on regulation of gravitropism. Even though numerous reports on gravitropism are published, the actual gravity receptor has not been identified yet. Auxin, ethylene, cytokinin and BRs have been the most explored hormones in relation to gravitropism but not much evidence has been accumulated regarding the participation of other phytohormones such as; Gibberellins (GAs), abscisic acid (ABA), jasmonates (JA), and salicylic acid (SA) in gravitropism.

### Auxin as an Essential Player in Root Gravitropism

**FIGURE 3:**
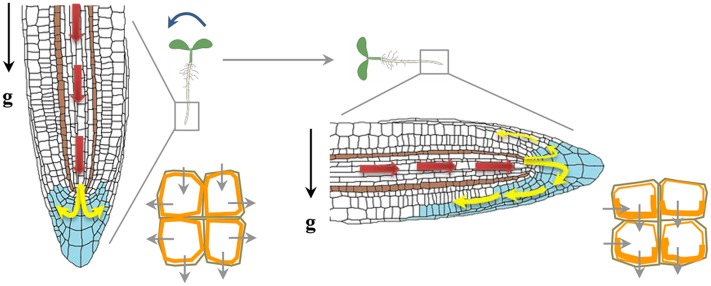
Auxin redistribution upon a gravity stimulus. Auxin distribution (blue) and direction of flow (depicted by arrows). Auxin from the shoot to the root tip (red arrows) is mediated by AUX1 and PIN2. Auxin flow is further distributed through the vascular tissue to the columella cells (yellow arrows). PIN3 is localized in the columella cells and gravity stimulus induces more PIN3 localization (orange) to the lower side of columella cells redirecting auxin flow to the lower side of the root (Friml et al., 2002). Asymmetric localization of PIN2 upon gravity reorientation too helps auxin flow at the lower side of gravistimulated root mainly directed towards the elongation zone, where it inhibits elongation at the lower side of the root and causes root bending.

Auxin was the earliest hormone to be identified with an implicated function during gravitropism, and for many years it dominated as the primary hormone regulating graviresponses. Auxin transport and response to auxin is pre-requisite for the development of tropic curvatures. Auxin, which is mainly synthesized in young shoot tissues, uses a cell-to-cell transport system that functions in the tip to base direction in shoots. When auxin reaches the root, it is transported through the central cylinder into root tip, where it adds to a pool of locally synthesized auxin, forming an auxin-maximum center that overlaps with the quiescent center and top layers of the root-cap columella. There, auxin is redistributed laterally to peripheral tissues, then transported basipetally through lateral-cap and epidermal cell files toward the elongation zone, where it inhibits cell elongation (**Figure 3**). Upon gravity reorientation, auxin accumulates in the lower region of the roots, as a result the cells of upper region elongate more and gravistimulated roots display a downward curvature. Modulation of auxin transport is therefore crucial for auxin redistribution and formation of an auxin gradient across a gravistimulated organ (Friml, 2003; Vanneste and Friml, 2009). The role of auxin transport in gravitropism was further understood by extensive studies examining the effect of various auxin transport inhibitors on the gravitropic response and also by showing that mutations in genes encoding key components of auxin transport machinery altered root gravitropism.

There are three types of transmembrane proteins that mainly transport auxin across the plasma membrane; (i) the transmembrane proteins AUXIN RESISTANT 1/LIKE AUX1 (AUX1/LAX), which operates as influx carriers to enable the transport of protonated auxin (Swarup and Péret, 2012); (ii) PIN-FORMED (PIN) proteins acting as auxin efflux carriers and (iii) members of the MULTIDRUG-RESISTANT (MDR)/P-GLYCOPROTEIN (PGP) family of ATP-binding cassette (ABC) transporters which are auxin efflux transporters. Mutation in AUX1 causes disruption of the basipetal auxin transport from the shoot tip to the root tip causing a reduction in the gravitropic root response of the mutant. Using fluorescent pH sensors, a marked increase in the surface pH on the lower side of a gravistimulated root was observed in WT but not in *aux1* mutant (Monshausen et al., 2011). This finding suggests that AUX1 is important in root gravitropism as the increase in root apoplastic pH results in more auxin in its ionic IAA^-^ form which is not permeable and requires AUX1 as a carrier (Swarup and Péret, 2012). Several lines of evidence strongly support a role of PIN auxin efflux carriers in gravitropism. In roots, PIN3, 4 and 7 are localized in columella cells (Sato et al., 2015). The PIN3 protein is expressed in upper S1 and S2 layers of the columella cells, whereas PIN7 protein is found in the S2 and S3 layers where they regulate auxin gradient immediately upon gravistimulation across the root cap (Kleine-Vehn et al., 2010). In the absence of PIN3, PIN7 expands its expression into the S1 layer and compensate for the loss of PIN3 (Kleine-Vehn et al., 2010). In vertically growing roots, these PINs show non-polar localization whereas upon gravistimulation, they are endocytosed and localized to the lower side of plasma membrane; thereby generating lateral auxin gradient across the root cap (Strohm et al., 2012). The PIN3 protein is recycled in an actin dependent manner (Friml et al., 2002). The involvement of PIN3 and PIN7 in gravitropism is substantiated by the fact that *pin3* and *pin7* mutants show gravitropic defects, and the *pin3 pin7* double mutant shows even stronger defects than their respective single mutants (Friml et al., 2002; Kleine-Vehn et al., 2010). The *pin2* mutant is agravitropic and the auxin distribution upon gravity stimulation is perturbed in *pin2* background (Luschnig et al., 1998). The polarity of PIN proteins is regulated by intracellular trafficking. Small GTPases (small G proteins) and GDP/GTP exchange factor (GEF) proteins regulate the root-ward localization of PINS. GNOM which is a GEF, modulate the activity of a small G-protein named ADP ribosylation factor (ARF) and affects the localization of PIN3 proteins (Strohm et al., 2012). BrefeldinA (BFA) inhibits trafficking of PIN3 by inhibiting GNOM, as a result PIN3 endocytosis continues but they get trapped in intracellular compartments (Kleine-Vehn et al., 2010). Engineering GNOM proteins in such a way that they become BFA insensitive (Geldner et al., 2003), lead to proper localization of PIN1 and PIN3, proper auxin gradients after gravistimulation and normal gravitropism even in presence of BFA (Geldner et al., 2003; Kleine-Vehn et al., 2010). Another gravitropism responsive gene identified by forward genetics approach is *SPIKE1 (SPK1).* SPK1 is a GEF for Rho-like GTPase from Plants 6 (ROP6). The *spk1* mutants show slow gravitropic response and decreased PIN2 levels at the plasma membrane, whereas overexpression of *ROP6* causes an increased gravitropic response (Lin et al., 2012). SPIKE1 binds to the inactive form of ROP6 and inhibit PIN2 endocytosis in roots (Lin et al., 2012). The polar localization of PIN protein is also dependent upon their phosphorylation status as use of kinase inhibitors has been shown to disrupt localization of PIN2 (Sukumar et al., 2009). Phosphorylation by PID kinase and dephosphorylation by a TYPE 2A PROTEIN PHOSPHATASE (PP2A), ROOTS CURL IN NAPHTHYLPHTHALAMIC ACID1 (RCN1) act antagonistically to each other and regulate auxin transport and gravitropism (Sukumar et al., 2009). The continuous recycling of PIN proteins helps in the dynamic control of polar localization and abundance at the plasma membrane which ultimately determines the rate and directionality of auxin flow (Baster et al., 2013). Recently, the R2R3-MYB type transcription factors FOUR LIPS (FLP) and its paralogue MYB88 has been reported to regulate transcript abundance of PIN3 and PIN7 in the gravity sensing cells of primary as well as lateral roots of Arabidopsis (Wang et al., 2015). The fact that most of the mutations in auxin transporters caused impaired root gravitropism strengthens the credit for auxin transport to play a more important role in gravitropism. However, the auxin gradient across the gravistimulated organ is transient and auxin asymmetry does not persist during the whole length of the gravitropic response. Such a temporal auxin asymmetry seems to be responsible for activation of auxin-responsive genes and subsequent downstream events leading to the bending response.

Analysis of gravitropic response of mutants defective in auxin-signaling provided additional support for the involvement of auxin response pathway during root gravitropism. Upon gravistimulation, many auxin-responsive genes are differentially regulated. Auxin is sensed by members of TRANSPORT INHIBITOR RESPONSE 1/AUXIN-RELATED F-BOX (TIR1/AFB) family of auxin receptors (Parry et al., 2009). AUXIN RESPONSE FACTORS (ARFs) family of transcription factors bind to auxin-responsive *cis*-acting elements (*AuxREs*) present in auxin induced genes (Remington et al., 2004). The AUXIN/INDOLE-3-ACETIC ACID INDUCIBLE (AUX/IAA) proteins are auxin induced nuclear localized short-lived repressor proteins. Under low auxin concentration, ARFs form dimers with AUX/IAAs, thereby shutting down transcription of auxin regulated genes. The repression of transcription by AUX/IAAs is also facilitated by TPLs (Szemenyei et al., 2008). Upon auxin accumulation, AUX/IAA is rapidly degraded executed via active E3 ligase SKP-Cullin-F-box^TRANSPORT INHIBITOR RESPONSE 1^ (SCF^TIR1^) complex and this process allows ARF-ARF dimerization and transcription activation of target genes (Leyser, 2002). Mutations in the AUX/IAA genes; *IAA3*, *IAA7*, *IAA17* and *IAA14* conferred abnormal gravitropic response in the hypocotyls and roots of their respective mutants *short hypocotyl 2* (*shy2*), *auxin resistant 2* (*axr2-1*), *axr3* and *solitary root* (*slr-1*) respectively (Leyser et al., 1996; Tian and Reed, 1999; Fukaki et al., 2002; Masson et al., 2002). The auxin-insensitive *tir1*, *axr6*, and *axr1* mutants also exhibited a reduced gravitropic response in roots (Masson et al., 2002). The *arf7* and *arf19* single mutants did not show gravitropic defects, but *arf7arf19* double mutant showed abnormal gravitropism in both hypocotyl and roots (Okushima et al., 2005). Furthermore, upon gravistimulation, the asymmetric auxin distribution leads to the expression of *ARF19.* Mutations within genes that affect activity and stability of auxin carriers also play a role in gravitropism; the *axr4* mutant showed gravitropic defects similar to that of *aux1* mutant. Similar to *aux1*, the *axr4* mutant’s gravitropic defect was rescued by application of naphthalene-acetic acid (NAA) but not by IAA or 2,4-dichlorophenoxy-acetic acid (2,4-D) (Masson et al., 2002). Later on, it was confirmed that AXR4, a protein present in endoplasmic reticulum, regulates localization of AUX1 and the agravitropic phenotype of *axr4* is caused due to defective AUX1 trafficking in the root epidermis (Dharmasiri et al., 2006). A small secretory peptide in Arabidopsis named GOLVEN (GLV) affects auxin transport by regulating distribution of PIN2 proteins (Whitford et al., 2012). *TIR2*, which encodes for TRYPTOPHAN AMINOTRANSFERASE a key enzyme in auxin biosynthesis, is also found to be differentially expressed on the lower sides of the roots upon gravistimulation (Yamada et al., 2009). The *tir2-1* mutant shows reduced gravitropism upon gravity reorientation (Yamada et al., 2009). In a recent study the early phase of root gravitropism involving auxin-induced ion signaling is regulated by CYCLIC NUCLEOTIDE-GATED CHANNEL 14 (CNGC14) which regulates rapid Ca^2+^ and pH changes in response to auxin treatment (Shih et al., 2015). Mutation in this gene leads to reduced auxin mediated gravitropic bending giving us a glimpse of evolutionary conserved auxin machinery which utilizes this module to accelerate gravitropic response (Shih et al., 2015). Further, an auxin-signaling mutant *patatin-related phospholipase-AI-1* (*pplaI-1*) showed increased root coiling response when grown on a non-penetretable agar medium tilted at 45° suggesting a role for auxin signaling in other root directional response such as root waving and coiling in Arabidopsis (Perrineau et al., 2016). In coming years, more investigation at genetic level will help clear the picture of role of auxin and its significance in controlling root gravitropism.

### Ethylene and its Role in Root Gravitropism

Besides auxin, ethylene is another phytohormone that has been widely investigated during regulation of gravitropism. Although ethylene is intimately involved in regulating growth at the cellular level, its influence on graviresponses might not be a direct one. Ethylene activates local auxin signaling pathway and regulates root growth by regulating auxin biosynthesis or by modulating the auxin transport (Růzicka et al., 2007). Exogenously applied ethylene could strongly inhibit elongation and curvature of gravistimulated roots. Ethylene also delayed the progression of asymmetric auxin distribution across the root upon gravity-stimulation (Buer et al., 2006). In addition, inhibition of ethylene synthesis or action also reduced gravicurvature in maize roots (Lee and Evans, 1990). The ethylene induced inhibition of gravitropism is not seen in *ethylene response1* (*etr1*) and *ethylene insensitive2* (*ein2*) mutants, this shows that an intact ethylene signaling is required for differential growth upon gravi-stimulation (Buer et al., 2006). Ethylene interacts with auxin to regulate root gravitropism. Mutations in the auxin influx protein AUX1 and in the auxin efflux carrier PIN2/ETHYLENE INSENSITIVE ROOT 1 (EIR1)/WAVY ROOTS6 (WAV6)/AGRAVITROPIC ROOT1 (AGR1) can cause insensitivity to ethylene as well (Pickett et al., 1990; Luschnig et al., 1998). Thus, ethylene insensitivity in the roots of *aux1* and *agr1*/*eir1*/*pin2*/*wav6* mutant plants may relate to the role of ethylene in regulating auxin transport and further pointing to the importance of the interactions between these two plant hormone signaling pathways. Similarly, roots of the *chirality and gravitropism* (*clg1*) mutant that were resistant to various auxins, also displayed a notable resistance towards ethylene (Ferrari et al., 2000). Ethylene could also suppress the formation of root loops, a gravity dependent growth response, in presence of high nutrient and Suc availability (Buer et al., 2003). The roots of *1-aminocyclopropane-1-carboxylic acid-related long hypocotyl 1* (*alh1*) mutant of Arabidopsis which was less sensitive to ethylene and auxin, displayed a faster response to gravity (Vandenbussche et al., 2003). In tomato, ethylene and auxin crosstalk has been shown to regulate root penetration into the soil (Santisree et al., 2011). The Arabidopsis mutant *root handedness 1* (*rha1*), a heat-shock factor, is resistant to ethylene and is perturbed in roots slanting, gravitropism and auxin physiology (Fortunati et al., 2008). It is also found that ethylene and gravity can also affect root skewing and waving in Arabidopsis (Oliva and Dunand, 2007). Additional evidence suggests that ethylene may be involved in the gravitropic response of roots via its effect on starch metabolism. Thus, exogenous ethylene reduced starch levels in Arabidopsis root columella cells and also the magnitude of gravitropic curvature (Guisinger and Kiss, 1999). Since amyloplasts are believed to be necessary for gravity sensing, these results may imply that ethylene accumulation can modify the gravity perception events. Ethylene biosynthesis enzyme; 1-aminocyclopropane-1-carboxylate (ACC) synthase (ACS) has also been shown to play a role in root gravitropism (Huang et al., 2013). The *acs7-1* loss of function mutant showed less sensitivity to the inhibition of root gravitropism by calcium chelator ethylene glycol tetra acetic acid (EGTA) (Huang et al., 2013). The *acs7-1* mutant was also less sensitive to inhibition of the gravity response by NPA which could be restored by application of ACC (Huang et al., 2013). Many plant organs show a transient burst of ethylene production when transferred from a vertical to a horizontal position. Altogether, plants have developed the ethylene pathway as a possible mode for coping with environment beneath and above the soil by helping the seedlings to modulate their differential growth. Based on previous and recent findings it is clear that ethylene plays a crucial role in gravitropic growth.

### Gibberellins and Its Role in Root Gravitropism

Gibberellins are prime regulators of cell elongation (Feng et al., 2008), therefore it is reasonable to speculate their involvement in gravistimulation responses. The GA signaling acts through GA-induced proteasomal degradation of repressor proteins of the DELLA family, thereby activating transcription of growth-promoting genes (Schwechheimer, 2008). GAs modulate gravitropism at least partly through transcriptional regulation of *IAA19*/*MSG2* also the agravitropism of *msg2-1* mutant was alleviated by inhibiting GA biosynthesis using paclobutrazol (PAC) (Gallego-Bartolomé et al., 2011). In a recent study, it was found that there is an asymmetric distribution of GA during root gravitropic growth (Löfke et al., 2013). GA also stabilizes PIN proteins by preventing their trafficking to the lytic vacuole, thereby stabilizing PIN2 at the lower side of the gravistimulated root. This promotes asymmetric auxin flow and distribution and gravitropic bending (Löfke et al., 2013). These findings provide an emerging picture for GAs to be a part of the complex network in regulating gravitropism and also strengthening auxin action during gravitropic responses.

### Abscisic Acid and Its Role in Root Gravitropism

Abscisic acid exerts mainly inhibitory effects on growth and development. Initial studies on the role of ABA in gravitropism were a little discouraging due to several reasons. Exogenously applied ABA does not inhibit rather promotes root growth, and the inhibitory effect is gained only at concentrations significantly higher than those thought to naturally occur. Also, roots of ABA-deficient plants obtained either by chemically inhibiting ABA synthesis or by specific mutations showed no altered response to gravity (Moore, 1990). When half of the root cap of vertically oriented maize roots was substituted with agar block containing ABA, it had little or no effect on curvature relative to that of controls having plain agar block (Lee et al., 1990). Another study for identification of endogenous growth regulators in graviresponding plant organ found no significant lateral asymmetry of endogenous ABA in root tips of *Zea mays* and *Vicia faba* (Mertens and Weiler, 1983). Since ABA plays a positive role in hydrotropism, its putative role in gravitropism is masked (Takahashi et al., 2002). Based on studies with various auxin and ABA mutants, it was hypothesized that ABA may serve as a regulator of auxin transport in root hydrotropic response and a similar interaction may also exist during root gravitropism (Eapen et al., 2005). Recently it was reported that exogenous ABA affects gravitropism by playing a role opposite to that of auxin and it acts as a negative regulator of the root gravitropic response in Arabidopsis (Han et al., 2009). ABA signaling in guard cell involves K^+^ efflux to regulate turgidity which in turn involves Phospholipase D (PLD) (Jacob et al., 1999). It was found that ectopic overexpression of *PHOSPHOLIPASE Dζ2* gene (*PLD*ζ*2*) enhances root gravitropism in Arabidopsis (Li and Xue, 2007). Similarly, ABA treatment and the *pld*ζ2 mutation together did not cause an additive effect with each other on root gravitropism suggesting that *PLD*ζ2 is involved in ABA signaling that suppresses root gravitropism (Taniguchi et al., 2010). In conclusion, combining these reports on role of ABA in root -gravitropism and -hydrotropism as well as its interactions with auxin, we may imply a probable role for ABA in mediating gravitropism-related responses.

## Cytokinins and Its Role in Root Gravitropism

Cytokinins are hormones that regulate cell division and development and play essential and crucial roles in various aspects of plant growth (El-Showk et al., 2013). Several studies have provided evidence for involvement of cytokinin in the gravitropic response. Since cytokinin has a negative regulatory role in root growth, it was suspected that it might also function as an inhibitor of tropic root elongation during gravity response. In roots, cytokinin is produced in the root cap cells, which regulate growth and gravitropism (Aloni et al., 2004). Lateral exogenous application of cytokinin through agar block to a vertically growing root induced bending towards the site of application (Aloni et al., 2004). These findings confirmed the inhibitory effect of cytokinin on root elongation during gravitropic bending (Aloni et al., 2004). When vertically growing Arabidopsis roots were gravistimulated, free cytokinin was radially transported and got asymmetrically distributed to the lower side of root cap within 30 min as detected by *ARR5::GUS* expression (Aloni et al., 2004).

Cytokinin may also interact with auxin machineries to regulate gravitropic response (Philosoph-Hadas et al., 2005). The *cytokinin induced root curling 1* (*ckrc1*) mutant of Arabidopsis which is allelic to *taa1* exhibits defective root gravitropic response which was rescued by exogenous application of auxin and increased resistance to cytokinin in primary root growth (Zhou et al., 2011). Depletion of endogenous cytokinins by overexpressing *CYTOKININ OXIDASE DEHYDROGENASE* (*CKX*) genes caused lateral expansion of the auxin maxima, i.e., from columella to lateral root cap (Pernisová et al., 2009). Further, cytokinin signaling defective mutant *ahk3* and cytokinin deficient *Pro35S:AtCKX* transgenic lines showed defects in both auxin redistribution as well as root gravitropic response (Pernisova et al., 2016). Upon gravistimulation, the AtCKX3 overexpression and *ahk3* mutant lines showed WT like pattern for PIN3 and PIN7 relocalization in columella while the *pin3pin4pin7* triple mutant did not show any defect in cytokinin sensitivity. On the other hand, depleting endogenous cytokinins via AtCKX overexpression caused alteration in cellular distribution of auxin influx carrier AUX1 suggesting that cytokinin largely targets AUX1-mediated auxin transport rather than PIN-mediated auxin transport to affect root gravitropism (Pernisova et al., 2016). In an interesting finding, role of cytokinin was established in cytokinin-induced root tip reorientation growth response which involves members of two-component system, i.e., ARABIDOPSIS HISTIDINE KINASEs (AHKs); and type-A and type-B RESPONSE REGULATORs (ARRs) (Kushwah et al., 2011). In conclusion, cytokinin seems to play a key regulatory role in root gravitropism. However, a close and complex interaction between cytokinin and auxin together with other hormones seems to take place in regulation of gravitropic growth.

### Brassinosteroid (BR) and Its Role in Root Gravitropism

Brassinosteroid (BRs) are regarded to be essential substances for growth and development in plants, and their occurrence has been demonstrated in all plant organs. Brassinolide (BL) was found to increase the gravitropic response of roots by increasing their sensitivity to IAA (Kim et al., 2000). BL can also stimulate the gravitropic response in maize roots via both ethylene dependent as well as independent mechanisms (Chang et al., 2004). In *Pisum sativum* roots, 24-epibrassinolide (EBL) treatment could reduce the average lag-time needed for gravitropic response initiation in a dose dependent manner (Amzallag and Vaisman, 2006). Further, treatment with steroid biosynthesis inhibitor clotrimazole could prevent the gravitropic response initiation which was partly rescued by EBL application (Amzallag and Vaisman, 2006). Exogenous application of BR promotes acropetal and basipetal auxin transport in Arabidopsis and *Brassica* roots (Li et al., 2005). BRs can also regulate gravitropic response of Arabidopsis roots by stimulating the activity of ROP2 GTPase, which mediates polar auxin transport, resulting in an increased gravitropic response (Li et al., 2005). However, both BL as well as IAA, at higher concentrations, antagonize the gravitropic curvature response induced by each other via modulating biosynthesis of the counterpart hormone (Kim et al., 2007). Like auxin, exogenous BR application could also affect actin cytoskeleton configuration and PIN2 localization patterns which in turn modulates auxin gradients maintenance and affect gravitropic responses in Arabidopsis roots (Lanza et al., 2012). These results show that BRs have a prominent role in controlling root gravitropic responses.

### Jasmonic Acid and Its Role in Root Gravitropism

Jasmonic acid is mostly studied in regulating plant defense but its function during plant growth and development is also fast emerging. There is very less information available in context of root gravitropism. As modulation of JA homeostasis as well as signal transduction can mimic auxin effects on root development, we assume it to have some effect on root gravitropic responses as well. In rice coleoptiles, the total content of JA is found to be increased upon gravity reorientation (Gutjahr et al., 2005). Also, a JA gradient is established opposite to the internal auxin gradient across the stimulated organ positively modulating gravitropic curvature. This JA-gradient in response to gravitropic stimulation is developed in an IAA-independent manner (Gutjahr et al., 2005). Interestingly, a JA-deficiency in rice mutant *hebiba* could not abolish its gravitropic response suggesting that JA might not be essential but could accelerate the gravitropic bending response (Gutjahr et al., 2005). In Arabidopsis, tryptophan conjugates of JA (JA-Trp) which act as IAA-antagonists can cause root agravitropism in a dose-dependent manner. The JA-Trp functions in a TIR1-dependent manner but are independent of COI1 (Staswick, 2009). In conclusion, JA might regulate root gravitropic responses via affecting auxin biosynthesis and gradient formation via modulating polar auxin distribution.

## Light as a Trigger for Change in Root Gravitropism

Light is an essential component for energy production and survival in plants. Light regulates nearly all stages of plant development on the basis of its quantity, quality and directionality. On the other hand, gravity is a constant stimulus providing plants with the critical information about its surroundings and thus guiding plant growth. Evidences have shown that light is required for triggering gravitropism in plants. The roots of *Vanilla planifolia* when exposed to light responded rapidly to the stimulus of gravity (Irvine and Freyre, 1961). In *Convolvulus arvensis* root when exposed to light showed positive orthogeotropic response. Red light enhanced the response and far-red-light exposure reversed the effect of red light (Tepfer and Bonnett, 1972). Different light spectrum is sensed by a variety of photoreceptors such as phytochromes, cryptochromes, phototropins, zeitlupes, and UVR8 (de Wit et al., 2016). There are few evidences in literature involving light mediated regulation of root movements. Roots also possess the phytochromes which sense R and FR light to mediate root elongation and directional growth (Correll and Kiss, 2005). The *phyB* and *phyAB* mutants showed delayed response toward gravity reorientation (Correll and Kiss, 2005). Phytochrome-A has been shown to play distinct role in red light-induced positive root phototropism (Kiss et al., 2003). Phytochrome-A also inhibits blue light mediated negative phototropism in Arabidopsis roots (Kiss et al., 2007). Light can also control root orthogravitropic response in maize via Ca^2+^/calmodulin-dependent protein kinase cascade (Lu and Feldman, 1997). Some evidence also suggests that these phytochrome also alters jasmonic acid response and sensitivity in roots (Costigan et al., 2011). In Arabidopsis, blue light photoreceptor PHOT1 is highly expressed in the root tissue (Mo et al., 2015). PHOT1 regulates the expression of PINOID kinase and PP2A phosphatase to mediate PIN3 polarization in the root thereby facilitating root bending (Zhang et al., 2013). Interestingly, PHOT1 is also found to regulate PIN2 localization through NPH3 proteins and blue light illumination was able to reduce gravitropic bending (Wan et al., 2012). Under microgravity conditions, blue light induced phototropism in roots was greatly reduced at 0.1g and masked at 0.3g and higher gravity levels displaying a novel response (Vandenbrink et al., 2016). PINOID kinases which are known to regulate auxin efflux carriers have been shown to negatively regulate root phototropism (Haga and Sakai, 2015). When Arabidopsis roots are illuminated with unilateral blue-light PID proteins are accumulated on the non-illuminated side of the root (Haga and Sakai, 2015). Flavonols in the transition zone have been shown to function as a positional signal interacting with hormonal and ROS pathways to regulate root directional growth to light (Silva-Navas et al., 2016). Upon root illumination, the flavonols accumulate towards the side exposed to light and causing differential growth promoting root movement away from light (Silva-Navas et al., 2016). The possible role of light in root gravitropism can further be strengthened by the fact that shoot phytochrome-B induces and stabilizes the expression of *ELONGATED HYPOCOTYL5 (HY5)* protein which as a shoot-to-root mobile signal regulates root growth and nitrate uptake (Chen et al., 2016). Later micrografting studies using Col-0 and *phyB-9* plants followed by subsequent illumination of grafted shoots suggested that root derived phyB regulates root gene expression (Lee et al., 2016). Since photoreceptors have a high sensitivity towards different lights, it is possible that a small level of stem piped light can bring significant changes in root architecture. It is now proven that not only light in the range of Far-Red and near Infra-Red is transmitted through the stems to roots but also light in the range of green to red is also effectively transmitted as a signal (Chen et al., 2016; Lee et al., 2016). Plants adapt to the changing environment by sensing the intensity and quality of light to regulate root growth and development and root gravitropism (Lee et al., 2016). Increased light fluxes can also modulate the levels of photosynthetically generated sugars that in turn can affect the directional movements in Arabidopsis such as root coiling, waving and deviation from vertical gravity vector (Singh et al., 2014a). In the event of recent developments, further identification of light induced mobile signals will help in finding new targets to modulate root gravitropism and architecture. A detailed analysis of vascular tissues and how they transduce light signals will be helpful in regulating plant architecture.

## Glucose as an Emerging Player in Root Gravitropism

Nutrient availability is a major factor controlling growth in a constantly changing environment. Plants, like other living organisms, need to maintain nutrient and energy homeostasis within cells and tissues for growth. They fulfil their energy requirement by fixing light into a metabolizable form via photosynthesis where carbohydrate (sugar) energy is utilized as fuel for growth and/or stored as reserve. Sugars are the prime carbon and energy source to build and fuel cells, and also acquired important regulatory functions in controlling metabolism, stress resistance, growth and development. Sugars also have an important signaling function and act like hormones in translating nutrient status to regulate growth and floral transition (Koch, 2004; Price et al., 2004; Rolland et al., 2006; Ramon et al., 2008; Smeekens et al., 2010; Lastdrager et al., 2014). Sucrose being the primary transport sugar, can be sensed as a signal either directly (Chiou and Bush, 1998); or via its hexose cleavage products such as glucose (Glc), UDP-glucose and fructose (Rolland et al., 2002; Price et al., 2004; Li et al., 2011). Glc being the second most abundant sugar in Arabidopsis has an important role to play in plant growth and development (Moore et al., 2003). Transcriptomics as well as genetic analyses have revealed that, in plants, many genes are regulated by Glc and its signaling networks (Price et al., 2004; Li et al., 2006). In recent years, a pivotal role of Glc in plant growth and development and key players in the Glc signaling network have been uncovered using Arabidopsis as the prime model system (Koch, 1996; Smeekens, 2000; Rolland et al., 2002; León and Sheen, 2003; Gibson, 2005; Rolland et al., 2006; Smith and Stitt, 2007). In plants, several sugar sensors exist which regulate growth and development. These sensors include HEXOKINASE 1 (HXK1) (Moore et al., 2003), REGULATOR OF G-PROTEIN SIGNALING 1 (RGS1) (Chen et al., 2003) and other sensors which respond to different sugars or sugar-derived metabolites, including trehalose-6-phosphate (Tre6P) and fructose etc. Thus, three distinct Glc signal transduction pathways operate in plants. One is the HXK1-dependent pathway governed by AtHXK1-mediated signaling function. The second is a HXK1-independent pathway where changes in gene expression are modulated by Glc but is independent of AtHXK1. The third is a glycolysis-dependent pathway that utilizes SUCROSE NON-FERMENTING RELATED KINASE1 (SnRK1)/TARGET OF RAPAMYCIN (TOR) pathway (Fu et al., 2014).

Sugar signaling pathway exhibits crosstalk with other response pathways such as those involved in light, phytohormones and stress responses. In plants, sugar and phytohormone signal cross-talks have been shown to modulate critical growth and developmental processes such as embryo establishment, seed germination, and early seedling growth and development (Gazzarrini and McCourt, 2001; Rolland et al., 2002; León and Sheen, 2003; Gibson, 2004, 2005; Rolland et al., 2006; Mishra et al., 2009; Kushwah et al., 2011; Gupta et al., 2012; Singh et al., 2014a,b; Gupta et al., 2015a,b). Apart from regulation of plant growth and development in general, there are few instances where sugar signals are reported to be controlling various directional growth responses in plants either independently or via interaction with other signals. For example, in maize, Glc from kernel to shoot becomes asymmetrically distributed in cortical tissue upon gravity-stimulation similar to radiolabelled IAA. This asymmetric distribution of Glc could also involve a lateral transport system as described for auxin (Momonoki, 1988). Glc- and Auxin signaling has also been found to interact extensively in regulating root gravitropism in Arabidopsis. The primary roots of Arabidopsis seedlings displayed a significant deviation from their vertical growth direction upon exogenous application of higher Glc concentrations (Mishra et al., 2009; Singh et al., 2014a). Also, the root gravitropic bending kinetics was significantly delayed in seedlings treated with high concentrations of Glc. Glc altered the root gravitropic response via both HXK1-dependent as well as HXK1-independent mechanisms (Singh et al., 2014b). Multiple phytohormone signaling components such as BR, cytokinin, ethylene and auxin work downstream to Glc to cause root deviation from vertical gravity vector response (Singh et al., 2014a,b). Glc promotes BRI1 mediated signaling by inhibiting the activity of Protein Phosphatase 2A (PP2A) (Singh et al., 2014b). Cytokinin and ethylene signals work further downstream and could antagonize this Glc response (Singh et al., 2014a). Exogenous Glc was able to alter rate of polar auxin transport and might utilize auxin transport machinery and further downstream components of auxin response pathway to modulate root gravitropic responses (Mishra et al., 2009; Singh et al., 2014b). Glc could also affect coiling and waving responses in Arabidopsis seedling root (Singh et al., 2014b). Glc interact synergistically with cytokinin to execute a novel root tip directional growth response in light grown Arabidopsis seedling (Kushwah et al., 2011). In Arabidopsis, TOR kinase dependent sugar/Glc signaling and energy homeostasis could also regulate root growth and development (Xiong et al., 2013). TOR-dependent signaling may interfere with auxin signaling to regulate root gravitropic response. The *TOR RNAi* seedlings were found to be defective in gravitropic bending response (Schepetilnikov et al., 2013). Also, chemical inhibition of TOR complex activity via Torin-1 pre-treatment could abolish the gravitropic bending response in Arabidopsis roots (Schepetilnikov et al., 2013). Rapamycin inhibits TOR kinase activity via FK506-binding protein 12 (FKBP12). Transgenic plants having functional FKBP12 in DR5::GUS background (DR5/BP12) showed loss of gravitropism upon treatment with rapamycin and another inhibitor of TOR, KU63794 (Deng et al., 2016). Recently, possible roles for TOR signaling complex in dictating various other directional growth responses of roots such as light escape in soil and salt avoidance have also been described (Yokawa and Baluška, 2015). These reports together reveal that a complex signaling network between sugar and plant hormones especially auxin response, light signals and energy balance homeostasis in cells of root apices operate to optimized directional growth behaviors in plant roots.

## Conclusion

Plants, being sessile organisms, use the coordinated action of several signaling pathways to grow and develop optimally in response to a changing environment. We know that light is an important factor in determining the directionality of plant growth. But gravity, a force that causes objects to fall and holds the planets in their orbits around the sun, is also critically important. Root directional growth and growth angle determines the area coverage in which it can capture water and nutrients and guides a plant to utilize nutrients that are unevenly distributed in soil. Plants have evolved to respond to different stimuli to help them orient to their best advantage. The growth and development of plants is mainly dependent on the platform set by the integrations of various signals such as light, gravity, nutrient, phytohormones etc. There are numerous examples of synergy, antagonism, and causal relationships among the different signaling pathways under various molecular and physiological processes, such as the control of cell expansion and divisions that define the architecture of vascular plants. Gravitropism is one of the major factors that determine root growth direction. Mechanism and control of gravi-response is a highly complex process which also involves several growth regulators. Recently introduced novel fluorescent pH indicator 8-hydroxypyrene-1,3,6-trisulfonic acid trisodium salt (HPTS) have enabled better understanding of asymmetric apoplast alkalization in gravistimulated roots and subsequent processes (Barbez et al., 2017). The identification of *negative gravitropic response of roots (ngr)* mutants in medicago and Arabidopsis, have provided further insight into the mechanism of root gravitropism (Ge and Chen, 2016). The loss of function of NGR genes (*AtNGR1*; *At1g17400*, *AtNGR2; At1g72490* and *AtNGR3; At1g19115*) reverses the root growth direction and the roots start to grow upwards against gravity vector (Ge and Chen, 2016). Further functional characterization of these mutants will help in connecting the dots of the model of gravitropism. New imaging techniques with rotating stages and tracking software such as Tip-Tracker has efficiently been used for imaging root growth and shown promises for the future of root gravitropic studies (von Wangenheim et al., 2017). Overall scenario suggests that in the phytohormonal hierarchy of gravitropic response, auxin homeostasis and cellular dynamics forms the converging point for most of the signals. However, individual roles of other phytohormones cannot be denied and it is the need of the hour to investigate the critical connecting links between these signals to strengthen our understanding towards the root tropic movements. With the availability of a plethora of mutants in Arabidopsis as well as other crop models and advanced molecular genetic studies, these complex interactions in various signaling pathways can be modulated to develop a range of improved plant varieties with optimal root growth behavior adapted towards a range of environmental challenges.

## Author Contributions

All authors have made intellectual contribution to the article, and approved it for publication. MS, AG, and AL conceptualized the article. MS and AG wrote the article and did final editing.

## Conflict of Interest Statement

The authors declare that the research was conducted in the absence of any commercial or financial relationships that could be construed as a potential conflict of interest.
